# Androgen receptor expression and response to chemotherapy in breast cancer patients treated in the neoadjuvant TECHNO and PREPARE trial

**DOI:** 10.1038/s41416-019-0630-3

**Published:** 2019-11-15

**Authors:** Isabell Witzel, Sibylle Loibl, Ralph Wirtz, Peter A. Fasching, Carsten Denkert, Karsten Weber, Hans-Joachim Lück, Jens Huober, Thomas Karn, Marion von Mackelenbergh, Frederik Marmé, Christian Schem, Elmar Stickeler, Michael Untch, Volkmar Müller

**Affiliations:** 1 Department of Gynecology, University Medical Center, Hamburg, Germany; 2 German Breast Group, Neu-Isenburg, Germany; 3 Stratifyer Molecular Oncology GmbH, Cologne, Germany; 4 Department of Gynecology and Obstetrics, University Hospital Erlangen, Comprehensive Cancer Center Erlangen-EMN, Friedrich-Alexander University Erlangen-Nuremberg, Erlangen, Germany; 5 Institute of Pathology, University Medical Center Charite Berlin, Berlin, Germany; 6 Practice of Gynecologic Oncology Hannover, Hannover, Germany; 7 Department of Gynecology and Obstetrics, University Medical Center Ulm, Ulm, Germany; 8 Department of Gynecology, University Medical Center Frankfurt, Frankfurt, Germany; 9 Department of Gynecology, University Medical Center-UKSH, Kiel, Germany; 10 Department of Gynecology and Obstetrics, University Medical Center Heidelberg, Heidelberg, Germany; 11 Mammazentrum, Krankenhaus Jerusalem, Hamburg, Germany; 12 Department of Gynecology and Obstetrics, University Medical Center Aachen, Aachen, Germany; 13 Department of Obstetrics and Gynecology, Helios Hospital Berlin-Buch, Berlin, Germany

**Keywords:** Predictive markers, Breast cancer, Translational research

## Abstract

**Background:**

The androgen receptor (AR) is discussed as a prognostic and/or predictive marker in breast cancer patients.

**Methods:**

AR mRNA expression was analysed by RT-qPCR in breast cancer patients treated in the neoadjuvant TECHNO (*n*  =  118, HER2-positive) and PREPARE trial (*n*  =  321, HER2-positive and -negative). In addition, mRNA expression of the AR transcript variants 1 (AR1) and 2 (AR2) was measured.

**Results:**

Regarding subtypes, high AR mRNA levels were frequent in HER2-positive (61.3%, 92/150) and luminal tumours (60.0%, 96/160) but almost absent in triple-negative tumours (4.3%, 3/69) (*p* < 0.0001). Overall, high AR mRNA levels were found to be associated with lower pathological complete remission (pCR) rates (OR 0.77 per unit, 95% CI 0.67–0.88, *p*  =  0.0002) but also with better prognosis in terms of longer disease-free survival (DFS) (HR 0.57, 95% CI 0.39–0.85, *p*  =  0.0054) and overall survival (OS) (HR 0.43, 95% CI, 0.26–0.71, *p*  =  0.0011). In the PREPARE trial, a survival difference for patients with high and low AR1 mRNA levels could only be seen in the standard chemotherapy arm but not in the dose-dense treatment arm (OS: HR 0.41; 95% CI 0.22–0.74 vs. HR 1.05; 95% CI 0.52–2.13; *p*  =  0.0459).

**Conclusions:**

We provide evidence that AR mRNA predicts response to chemotherapy in breast cancer patients.

## Background

Androgen receptor (AR) is expressed in normal breast epithelial cells and in ~70–90% of invasive breast carcinomas.^1^ Alternative splicing of the AR gene results in multiple transcript variants encoding different isoforms. The AR consists of a full-length 110 K AR protein (AR1) and an 87 K N-terminally truncated AR isoform (AR2). The distribution of both AR isoforms varies in different human tissues^2^ and their role in breast cancer has not been studied in detail so far.

AR is frequently co-expressed with the oestrogen receptor (ER) and progesterone receptor,^3^ but is less frequently expressed in HER2-positive tumours.^4,5^ The emerging role of AR in breast cancer patients is due to results supporting the prognostic value of AR in both ER-positive and ER-negative tumours.^6–10^ As a relevant percentage of triple-negative breast cancers also express AR, it has been identified as a potential new therapeutic target in this subset of patients with limited therapeutic options.^4^ In contrast to smaller studies in which no effect of AR expression in HER2-positive breast cancer was observed, a current meta-analysis reported that higher AR mRNA expression levels were found to be associated with improved overall survival in both uni- and multivariate analyses also for women with HER2-enriched breast cancer.^11^

Although most publications discuss AR as a prognostic marker in breast cancer patients,^6,9,12,13^ some reports also found an association between AR and therapy response. First, AR was assumed to be a predictive marker for response to endocrine treatment in breast cancer patients.^14,15^ Then, AR was evaluated in ER-positive tumours and a prognostic role of AR could be basically seen in chemo-endocrine treated patients.^7^ Additionally, Park et al. described no effect of high AR expression levels on chemotherapy benefit in ER-positive patients, but concluded that patients with low AR expression could be ideal candidates for chemotherapy treatment.^16^ Our group has subsequently shown that although AR predicts response to adjuvant chemotherapy rather than to endocrine treatment, the worst response rates were observed in patients with low AR expression.^17^ According to data from the neoadjuvant Gepartrio trial, Loibl et al. also reported that low AR expression determined by immunohistochemistry was associated with shorter disease-free and overall survival in 673 patients receiving chemotherapy with TAC.^18^

Despite this published data, the biological role of the AR expression in breast cancer is still not clear. Up to now, no consequences for therapy decisions based on AR expression in breast cancer therapy could be drawn. Moreover, the potential role of the two isoforms in the context of current therapeutic strategies has not been defined.

In our previous study, we could show that low AR mRNA was a predictor for shorter survival in breast cancer patients receiving adjuvant chemotherapy.^17^ The aim of this study was to validate these findings in a defined therapeutic context and to investigate the androgen receptor (AR) and in addition its two isoforms (AR1 and AR2) in breast cancer patients receiving neoadjuvant chemotherapy in two trials: the TECHNO trial (HER2-positive patients) and the PREPARE trial (HER2-positive and -negative patients).

## Methods

### TECHNO trial

The TECHNO trial was a multicentre, prospective, open-label, phase II clinical trial investigating neoadjuvant chemotherapy plus trastuzumab in HER2-positive breast cancer patients.^19^ Patients (*n* = 217) received four 3-week cycles of epirubicin 90 mg/m^2^ and cyclophosphamide 600 mg/m^2^ followed by the combination of four 3-week cycles of paclitaxel 175 mg/m^2^ with trastuzumab 6 mg/kg every 3 weeks (8 mg/kg as loading dose) (EC → Pac + trastuzumab) followed by surgery. Trastuzumab was continued as single agent postoperatively to complete 1 year of treatment. The primary endpoint was pathological complete remission (pCR), defined as absence of invasive breast cancer in the breast and axillary lymph nodes in all surgically excised specimens. Eligible patients had confirmed HER2-overexpressing primary breast cancer defined as a 3+ staining intensity by immunohistochemistry (IHC) using the DAKO HerceptTest (DAKO, Glostrup, Denmark) or a 2 + staining intensity centrally confirmed for HER2 gene amplification by fluorescent in situ hybridisation (FISH; PathVision Abbott; Abbott Park, IL). Tumours were either ≥2 cm based on clinical or ultrasound assessment or were diagnosed clinically as inflammatory breast cancer.

### PREPARE trial

The PREPARE trial was a multicentre, prospective, open-label, phase II clinical trial investigating a standard chemotherapy treatment consisting of neoadjuvant four 3 week cycles of epirubicin 90 mg/m(2) plus cyclophosphamide 600 mg/m(2) followed by four 3 week cycles of paclitaxel 175 mg/m(2) (EC → T), (*n* = 370), versus a dose-dense treatment arm with epirubicin 150 mg/m(2) followed by paclitaxel 225 mg/m(2) with pegfilgrastim support every two weeks followed by CMF (cyclophosphamide 500 mg/m(2), methotrexate 40 mg/m^2^, fluorouracil 600 mg/m(2)) on days 1 and 8 every 2 weeks (E(dd) → T(dd) → CMF), every 28 days (*n* = 363). Patients were randomly allocated to either simultaneous darbepoetin alfa (DA) (*n* = 356) or none (*n* = 377). Primary endpoint was pCR.^20^

### Assessment of AR mRNA Expression by RT-qPCR

Tumour specimens were assessed by RT-qPCR as previously described.^2^ In short, for RNA extraction from formalin-fixed, paraffin-embedded tissue, a single 10-μm curl was processed according to a commercially available bead-based extraction method (Xtract kit; STRATIFYER Molecular Pathology GmbH, Cologne, Germany). RNA was eluted with 100 μl of elution buffer. DNA was digested, and RNA eluates were then stored at −80 °C until use. Primers for the AR transcript variant 1 (AR1; RefSeq NM_000044.3) and transcript variant 2 (AR2; RefSeq NM_001011645.2) were designed. The mRNA expression levels of the genes of interest (GOI), AR, AR1, AR2 and ESR1as well as the reference gene (REF) CALM2 were determined by RT-qPCR, which involves the reverse transcription of RNA and subsequent amplification of cDNA executed successively in a one-step reaction. Each patient sample or control was analysed in triplicate in a Siemens Versant PCR System (Siemens, DE) according to the following protocol: 5 min at 50 °C and 20 s at 95 °C followed by 40 cycles of 15 s at 95 °C and 60 s at 60 °C. Forty amplification cycles were applied, and the cycle threshold (CT) values of the four GOIs and the REF gene for each sample were estimated as the median of the triplicate measurements. These were then normalised against the median expression levels of the REF gene using the 40-ΔCT method to ensure that the gene expression obtained by the test corresponds to the normalised log2 mRNA expression levels:

$${\it{\Delta {\mathrm{CT}}}}\left( {{\it{{\mathrm{GOI}}}}} \right)\,{\it{ = {\mathrm{40}} \, -\, }}\left( {{\it{{\mathrm{CT}}}}\left[ {{\it{{\mathrm{GOI}}}}} \right]{\it{\, - \, {\mathrm{CT}}}}\left[ {{\it{{\mathrm{REF}}}}} \right]} \right){\it{.}}$$

### Molecular subtype by RT-qPCR

In order to define molecular subtypes of the tumours, an additional RNA-based analysis with Mammatyper® was started. The MammaTyper® is a molecular in vitro diagnostic tool for the assessment of the expression levels of the four cancer biomarkers (HER2, ERα, PR and Ki-67) that are required for clinical management of breast cancer patients in clinical practice. We measured mRNA transcripts of the corresponding genes (ERBB2, ESR1, PGR and MKI67) with MammaTyper® in RNA samples retrieved from FFPE tissue as described before.^2,21^

### Study cohort

Of the initial cohort of patients in both trials (Intention-to-treat (ITT)-population *n* = 950), 477 tumour samples could be analysed (50.2%). Statistical analyses were performed on those 439 patients for whom at least one gene expression (AR, AR1, AR2 or ESR1) was available after quality control. In order to define molecular subtypes of the tumours, an additional independent RNA-based analysis with Mammatyper® was performed as described above. Table 1 contains a more detailed overview. The MammaTyper classifies samples into six molecular subtypes, some of which had only very few patients in our analysis set. For meaningful statistical analysis, we combined some of the classes (luminal A and B to luminal, luminal HER2-enriched to HER2-positive).

**Table 1:** Availability of tumour material in the study cohort

Patients (number)	TECHNO	PREPARE	Total
In study (intention-to-treat set)	217	733	950
expression data available	118	321	439
With AR available	113	305	418
With AR Isoform 1 available	117	319	436
With AR isoform 2 available	68	175	243
With ESR1 available	118	321	439
With MammaTyper^®^ available	103	276	379

There was no relevant imbalance between the whole study cohort and the subgroup of patients in our analysis regarding clinical and histopathological parameters (data not shown). 118 patients of the cohort (26.9%) participated in the TECHNO trial and 321 patients (73.1%) in the PREPARE trial. Median age of the patients was 48 years (range 25–67). Regarding tumour subtypes, 42.2% of patients (*n* = 160) had luminal, 39.6% (*n* = 150) HER2-positive and 18.2% (*n* = 69) triple-negative tumours. 47.4% of patients (*n* = 198) had node-positive disease, 15.7% of patients (*n* = 69) achieved a pCR. Detailed patient characteristics of the study cohort are shown in Table 2.

**Table 2:** Clinical and histopathological characteristics of all patients

	Cohort (analysed)	
	Number of cases	Total (%)
Study		
TECHNO	118	26.9
PREPARE	321	73.1
Age		
Median (years)	48	
Range (years)	25–67	
Grade		
Low (G1&2)	217	55.0
High (G3)	178	45.1
Unknown	44	
Nodal status		
Negative	220	52.6
Positive	198	47.4
Unknown	21	
Oestrogen receptor status		
Negative	161	41.3
Positive	229	58.7
Unknown	49	
Progesterone receptor status		
Negative	204	52.4
Positive	185	47.6
Unknown	50	
Subtype (MammaTyper^®^, combined)		
Luminal	160	42.2
HER2-positive	150	39.6
Triple-negative	69	18.2
Unknown	60	
Chemotherapy		
ddE-ddPAC (PREPARE)	150	34.2
EC-PAC (PREPARE)	171	39.0
EC-PAC-Trastuzumab (TECHNO)	118	26.9
pCR		
No	370	84.3
Yes	69	15.7

### Statistical analysis

AR, AR1, AR2 and ESR1 were analysed as continuous variables and in addition were dichotomised using the median as cut-off as described before.^22^

Pathological complete remission (pCR) was defined as no evidence of invasive or non-invasive cells in tissue of breast and lymph nodes. Disease-free survival (DFS) was computed from the date of study inclusion to the date of first metastasis or recurrence. Overall survival (OS) was computed to the date of death.

Dichotomised biomarkers were compared to binary variables by Fishers exact tests, to multi-level categorial variables by Chi-square tests and to continuous variables by Wilcoxon tests. For the clinical endpoint pCR as dependent variable logistic regression models were constructed, endpoints DFS and OS were analysed by Cox regression models. Univariate and multivariate regression models adjusting for subtype (luminal vs HER2-positive vs triple-negative), tumour size (T1–2 vs T3-4), nodal status (N0 vs N +), grading (G1-2 vs G3) and age (continuous) were built. Survival curves were compared with the logrank test.

All tests were performed at a significance level of *p* = 0.05 (two-sided).

The analysis was performed according to the REporting recommendations for tumour MARKer prognostic studies (REMARK) criteria on reporting of biomarkers.^23^

## Results

Given that the mRNA expression patterns of AR1 and AR2 were correlated in the nonparametric spearman correlation (rs: 0.6493, *P* < .0001) we report results exclusively for AR1. Furthermore, AR2 had no additional prognostic information.

### Androgen receptor expression and clinical variables

A positive association between AR1 and oestrogen receptor status (immunohistochemistry) (*p* < 0.0001) and progesterone receptor status (*p* < 0.0001) (Table 3) was observed. We found an inverse association between AR1 and grading (*p* < 0.0001) and pathological complete remission (pCR) (*p* = 0.0670). Regarding subtypes, high AR1 expression was more frequent in HER2-positive (62.0%, 93/150) and luminal tumours (65.0%, 104/160) whereas high AR1 expression was almost absent in triple-negative tumours (4.3%, 3/69) (*p* < 0.0001). No association between AR1 mRNA levels and nodal status, tumour size or age could be found (Table 3).

**Table 3:** Patients’ and histopathological characteristics according to AR mRNA expression and its two isoforms (AR-1 and AR-2) above and below the median

	AR -1		
	AR low (*n*)	AR high (*n*)	*p*-value
All	208	228	
Study			
TECHNO	53	64	0.5890
PREPARE	155	164	
Age			
Median (years)	49	48	0.2817
Range (years)	26–65	25–67	
Grade			
Low (G1 and 2)	76	139	<0.0001
High (G 3)	108	70	
Tumour size			
cT1 and cT2	150	177	0.7241
cT3 and cT4	45	48	
Nodal status			
Negative	107	111	0.4928
Positive	90	107	
Oestrogen receptor			
Negative	108	52	<0.0001
Positive	76	152	
Progesterone receptor			
Negative	124	79	<0.0001
Positive	60	124	
HER2			
Positive	67	82	0.4745
Negative	131	137	
Subtype			
Luminal	56	104	<0.0001
HER2-positive	57	93	
Triple-negative	66	3	
pCR			
Yes	40	29	0.0670
No	168	199	

### Androgen receptor expression and pCR

In the entire cohort, higher AR1 expression levels showed a significant association with lower pCR rates (OR per unit 0.77, 95% CI 0.67–0.88, *p* = 0.0002) in univariate analysis while not significant in a multivariate model (Table 4). This association with pCR could not be seen in the TECHNO trial but in the PREPARE trial in which high AR1 expression levels were associated with lower pCR rates (OR 0.66, 95% CI 0.55–0.78, *p* < .0001). In accordance, a non-significant trend to lower pCR rates in patients with high AR1 expression was observed in the triple-negative subgroup (OR 0.74, 95% CI 0.49–1.12, *p* = 0.1546) compared with the HER2-positive subgroup (OR 1.40, 95% CI 0.95–2.06, *p* = 0.0920) (Table 4). High ESR1 expression was associated with lower pCR rates (OR 0.73, 95% CI 0.65–0.82, *p* < 0.0001) in univariate analysis, even in the subgroup of patients with luminal tumours (OR 0.60, 95% CI 0.40–0.91, *p* = 0.0149), but not in multivariate analysis (data not shown).

**Table 4:** Logistic regression for AR1 mRNA and pathological complete remission rates (ypT0 and ypN0) (univariate and multivariate analysis)

AR1 mRNA	Univariate				Multivariate			
	Odds ratio	95% CI	*p*-value	*n*	Odds ratio	95% CI	*p*-value	*n*
All patients	0.77	0.67–0.88	0.0002	418	1.01	0.76–1.34	0.9305	323
TECHNO	1.02	0.75–1.39	0.8811	117	1.44	0.89–2.35	0.1369	95
PREPARE	0.66	0.55–0.78	<0.0001	319			^a^	
Luminal	0.81	0.50–1.29	0.3652	160			^a^	
HER2-positive	1.37	0.92–2.04	0.1245	150	1.35	0.86–2.11	0.1918	134
Triple-negative	0.74	0.49–1.12	0.1546	69			^a^	

^a^Calculation not valid

### Androgen receptor expression and survival

To address the impact of AR mRNA expression levels on prognosis, we compared the risk of relapse among all patients, and within different breast cancer subtypes. Overall, high AR1 mRNA levels compared to low AR1 mRNA levels were found to be associated with better prognosis in terms of DFS (HR 0.76, 95% CI 0.56–1.04, *p* = 0.0860) and OS (HR 0.60, 95% CI 0.40–0.89, *p* = 0.0103) in univariate analysis and in multivariate analysis (DFS: HR 0.62, 95% CI 0.41–0.92, *p* = 0.0173; OS: HR 0.50, 95% CI, 0.30–0.83, *p* = 0.0080) (Tables 5 and 6). Within different subgroups, no significant association between AR1 mRNA and survival could be seen.

**Table 5:** Disease-free survival for AR1 mRNA (above vs. below the median) (univariate and multivariate Cox regression analysis)

AR1 mRNA	Univariate				Multivariate			
	Hazard ratio	95% CI	*p*-value	*n*	Hazard ratio	95% CI	*p*-value	*n*
All patients	0.76	0.56–1.04	0.0860	431	0.62	0.41–0.92	0.0173	322
TECHNO	0.82	0.45–1.48	0.5135	117	^a^		^a^	
PREPARE	0.73	0.51–1.06	0.1004	314	0.58	0.35–0.94	0.0282	227
Luminal	0.89	0.51–1.57	0.6905	159	0.66	0.36–1.21	0.1772	136
HER2-positive	0.65	0.39–1.08	0.0980	148	0.64	0.36–1.13	0.1231	133
Triple-negative	^a^						^a^	

^a^Calculation not valid

**Table 6:** Overall survival for AR1 mRNA (below and above the median) (univariate and multivariate Cox regression analysis)

AR1 mRNA	Univariate				Multivariate			
	Hazard ratio	95% CI	*p*-value	*n*	Hazard ratio	95% CI	*p*-value	*n*
All patients	0.60	0.40–0.89	0.0103	413	0.50	0.30–0.83	0.0080	322
TECHNO	0.57	0.25–1.28	0.1723	117	^a^		^a^	
PREPARE	0.61	0.39–0.95	0.0300	314	0.43	0.23–0.81	0.0092	227
Luminal	0.69	0.35–1.37	0.2867	159	0.48	0.22–1.01	0.0544	136
HER2-positive	0.52	0.25–1.04	0.0652	148	0.60	0.27–1.32	0.2047	133
Triple-negative	^a^						^a^	

^a^Calculation not valid

For other markers, only in luminal patients and only in univariate analysis, high ESR1 expression was associated with better DFS (HR 0.76, 95% CI 0.63–0.92, *p* = 0.0043; data not shown).

### Androgen receptor expression and treatment

**Fig. 1:**
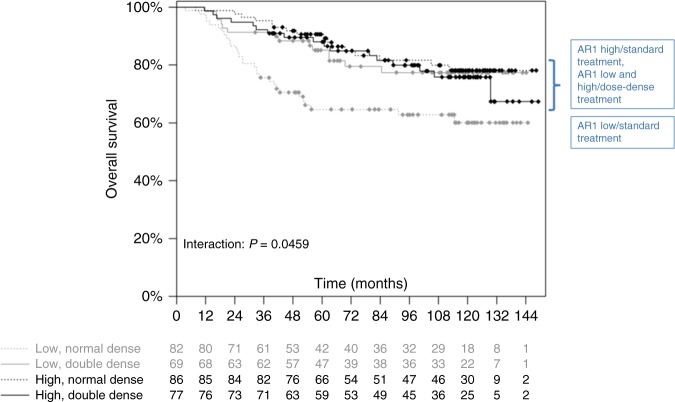
Overall survival in patients with high and low AR1 mRNA levels receiving dose-dense or standard chemotherapy treatment

Next, we hypothesised that the worse prognosis of patients with low AR1 mRNA expression could be modified by treatment. In the PREPARE trial, a dose-dense treatment arm was compared with a standard treatment arm. Regarding overall survival, patients with high AR1 mRNA levels receiving the standard treatment had better survival compared to patients with low AR1 mRNA levels (HR 0.41 (95% CI 0.22–0.74) while for patients with dose-dense treatment the survival did not differ depending on the AR1 level (HR 1.05 (95% CI 0.52–2.13), interaction *p* = 0.0459, Fig. 1).

## Discussion

Our data show that AR expression is a prognostic marker in chemotherapy-treated breast cancer patients. In our cohort, measuring the isoform AR1 could be of interest for the prediction of therapy response to dose-dense treatment.

In patients that did not receive any systemic treatment, we had already shown that the AR had no additional prognostic information.^17^ In contrast, in chemotherapy-treated patients, low AR mRNA expression was associated with shorter event-free survival.

Here, we could show that the group of patients with low AR1 mRNA levels also had worse survival rates compared with patients with high AR1 mRNA levels. But the worse prognosis might be overcome by dose-dense chemotherapy treatment in those patients with low AR1 mRNA levels. In line with data from bladder cancer, we could show that AR1 mRNA expression is the isoform, which gives further prognostic information in breast cancer patients.^22^

A potential drawback of our study is its retrospective nature and assessment of a biomarker that was not prospectively defined. We had AR data in 44.0% of patients (*n* = 418/950) of the whole study cohort. In addition, AR could be determined only on RNA level since additional paraffin-embedded tissue was not available from most patients. Therefore, a correlation between immunohistochemical analysis and mRNA levels could not be examined. Regarding AR positivity, immunohistochemical analysis reveals AR positivity in 65–80% of breast cancer patients.^9^ However, for AR, no uniform staining and scoring system is established and quantification is difficult.

In addition, in prostate cancer, no correlation between staining intensity and mRNA expression of the AR protein could be observed.^24^ Therefore, quantitative measurement by mRNA as applied in our study could be a valid alternative. Androgen receptor-targeted treatments for breast cancer are in development and have shown promising preliminary results.^25–28^ One direction in preclinical and clinical research is the use of AR antagonists in triple-negative breast cancer^25,26,28–30^ but up to now, no reliable biomarker has been identified to predict response. It is of interest that in our study very few patients with triple-negative breast cancer had high AR1 mRNA levels whereas in immunohistochemical studies AR-positivity rates of 12–32% in triple-negative tumours were described. This discrepancy might be one possible explanation why AR evaluation by immunohistochemistry does not predict response to AR-directed therapies.

In addition to the prognostic role of AR in breast cancer patients, there is growing evidence that AR predicts response to chemotherapy treatment rather than to endocrine treatment with the worst response rates in patients with low AR expression. According to data from the neoadjuvant Gepartrio trial, Loibl et al. reported that low AR expression determined by immunohistochemistry was associated with shorter disease-free and overall survival in 673 patients receiving chemotherapy with TAC.^18^ In this trial, AR-positive tumours had lower pCR rates (12.8 vs. 25.4%) and AR expression added independent predictive information for pCR.^18^ Within the non-pCR subgroup, AR positivity selected a group with a significant better survival but not within the pCR group.^18^ Interestingly, also in our cohort, patients with low AR1 mRNA levels had a higher probability to achieve a pCR than those with high AR1 mRNA levels. Despite this fact, patients with low AR1 mRNA levels had worse survival rates. However, we have to admit that the pCR rate of 15.7% in our study cohort is lower compared to pCR rates reached in recent neoadjuvant trials. This difference might be explained in part by the high number of patients with luminal tumours in our cohort who have lower pCR rates. In addition, high AR1 mRNA levels were rare in patients with triple-negative breast cancer who, on the other hand, have higher pCR rates.

To our knowledge, we are the first to document that the worse prognosis of patients with low AR1 mRNA levels might be compensated to a certain degree by dose-dense chemotherapy treatment. Survival differences could be mainly seen regarding overall survival rates whereas no significant interaction could be seen for AR1 mRNA levels, chemotherapy treatment and disease-free survival.

There is increasing evidence that dose-dense chemotherapy is beneficial in breast cancer patients.^31,32^ Currently node-positivity often guides the decision towards dose-dense treatment. We could show that AR1 mRNA may add information in the decision for or against dose-dense chemotherapy treatment, which would have to be validated in further studies.

In conclusion, we provide evidence that there seems to be an interaction between AR expression and chemotherapy-responsiveness in breast cancer patients. Determination of the isoform AR 1 might deliver additional information for the prediction of therapy response to dose-dense chemotherapy treatment.

## Supplementary information

Suppl. Table 1

## Data Availability

The datasets used during the present study are available from the German Breast Group, Neu-Isenburg, Germany on reasonable request.
