# Supplementary material for: Androgen receptor expression and response to chemotherapy in breast cancer patients treated in the neoadjuvant TECHNO and PREPARE trial
Source: Br J Cancer. 2019 Nov 15;121(12):1009–15. doi: 10.1038/s41416-019-0630-3 (PMC6964685; doi:10.1038/s41416-019-0630-3)
Supplement: Supplementary file 1 — Suppl. Table 1 [file 41416_2019_630_MOESM1_ESM.docx]

Supplementary Table 1: Study centres participating in the TECHNO and PREPARE trial with local ethics committees

- Berlin, Germany
- Bergisch-Gladbach, Germany
- Ebersberg, Germany
- Erlangen, Germany
- Esslingen, Germany
- Freiburg, Germany
- Hamburg, Germany
- Hamm, Germany
- Hannover, Germany
- Kassel, Germany
- Mainz, Germany
- Munich, Germany
- Rosenheim, Germany
- Ulm, Germany
- Wiesbaden, Germany
- Wuppertal , Germany
